# *MAPK* and *ERK* polymorphisms are associated with PCOS risk in Chinese women

**DOI:** 10.18632/oncotarget.22153

**Published:** 2017-10-27

**Authors:** Lingmin Hu, Yiting Zhang, Li Chen, Wei Zhou, Yong Wang, Juan Wen

**Affiliations:** ^1^ Department of Reproduction, The Affiliated Changzhou Maternity and Child Health Care Hospital of Nanjing Medical University, Changzhou 213003, China; ^2^ State Key Laboratory of Analytical Chemistry for Life Science, Jiangsu Key Laboratory of Molecular Medicine, Medical School of Nanjing University, Nanjing 210093, China; ^3^ Nanjing Maternity and Child Health Care Institute, Nanjing Maternity and Child Health Care Hospital, Obstetrics and Gynecology Hospital Affiliated to Nanjing Medical University, Nanjing 210004, China; ^4^ State Key Laboratory of Reproductive Medicine, Nanjing Maternity and Child Health Care Hospital, Obstetrics and Gynecology Hospital Affiliated to Nanjing Medical University, Nanjing 210004, China

**Keywords:** variants, MAPK/ERK, RegulomeDB, polycystic ovarian syndrome

## Abstract

In this case-control study, we analyzed the association between eight RegulomeDB-annotated single nucleotide polymorphisms (SNPs) in the *MEK1*, *MEK2*, *ERK1* and *ERK2* genes and polycystic ovarian syndrome (PCOS). Logistic regression analysis demonstrated that *MEK1* rs12050732 (OR = 1.29 [95%CI: 1.06-1.58], *P* = 0.012), *ERK2* rs2266966 (OR = 0.81 [95%CI: 0.67-0.99], *P* = 0.040) and *ERK2* rs5999521 (OR = 0.66 [95%CI: 0.51-0.86], *P* = 0.002) were associated with PCOS risk without adjusting for age and body mass index. Moreover, PCOS risk increased with allele dosage when these three polymorphisms were combined (*P*_trend_ = 0.001). These findings suggest that genetic variants in key *MAPK* and *ERK* genes contribute to PCOS risk in Chinese women.

## INTRODUCTION

Polycystic ovarian syndrome (PCOS) is a common endocrine syndrome associated with oligo-ovulation or anovulation among women of reproductive age [1, 2]. PCOS is characterized by elevated serum luteinizing hormone (LH) and testosterone (T) levels, although the serum follicle-stimulating hormone (FSH) levels are normal [3]. PCOS is also associated with increased risk for metabolic syndromes like insulin resistance, obesity and type 2 diabetes [2, 4, 5]. Both environmental and genetic factors contribute to the complex pathophysiology of PCOS. Candidate gene studies and genome-wide association studies (GWAS) have demonstrated that genetic components play an important role in PCOS susceptibility [6, 7].

MAPK/ERK signaling pathway includes mitogen-activated protein kinases (MAPKs), like extracellular signal-regulated kinases (ERKs), which integrate multiple biochemical and environmental signals through phosphorylation cascades. The MAPK/ERK signaling pathway regulates a wide variety of cellular processes such as proliferation, differentiation, transcriptional regulation and differentiation [8, 9]. The upstream core MAPKs such as MEK1 and MEK2 phosphorylate and activate downstream effector kinases, ERK1 and ERK2 [10]. The MAPK/ERK signaling pathway is associated with steroid biosynthesis in ovarian granulosa cells [11, 12]. Aberrant MAPK/ERK signaling contributes to metabolic signaling defects and excessive ovarian androgen production in women with PCOS [13, 14]. As the above, there is increasing evidence that MAPK/ERK pathway genes are associated with PCOS risk.

Functional annotation of GWAS data has shown that single nucleotide polymorphisms can be useful biomarkers for various human diseases [15, 16]. RegulomeDB is a database that integrates regulatory information from ENCODE and other data sources and is a powerful tool that shows functional SNPs in specific chromosomal regions [17, 18]. It has a scoring system from 1 to 6 for the annotated data on methylation, chromatin structure, protein motifs and binding. The lower score indicates high probability for a variant to be located in a functional region.

Several studies have used RegulomeDB to identify causal polymorphisms associated with human diseases such as lung cancer, colorectal cancer and so on [19–21]. In this case-control study, we analyzed PCOS risk with *MAPK/ERK* gene polymorphisms that are annotated in RegulomeDB.

## RESULTS

Table 1 shows clinical pathological characteristics of PCOS patients and controls in accordance with 2003 Rotterdam PCOS diagnose criteria. PCOS patients showed higher LH and T levels as well as body mass index (BMI) than control subjects.

**Table 1:** Clinical pathological parameters in PCOS and control subjects

Parameters	Case	Control	T-test	*P*
Age	27.36±5.27	30.68±3.66	9.030	<0.001
BMI	22.84±3.74	21.41±3.08	-5.200	<0.001
FSH (IU/L)	6.41±2.67	7.33±2.56	4.266	<0.001
LH (IU/L)	13.27±8.22	5.10±3.36	-14.933	<0.001
E2 (pg/ml)	66.94±48.52	54.34±39.37	-3.347	<0.001
T (ng/ml)	2.79±7.69	1.09±1.01	-3.428	<0.001

BMI: body mass index; FSH: follicle-stimulating hormone; LH: luteinizing hormone; E2: estradiol; T: total testosterone.

**Figure 1:**
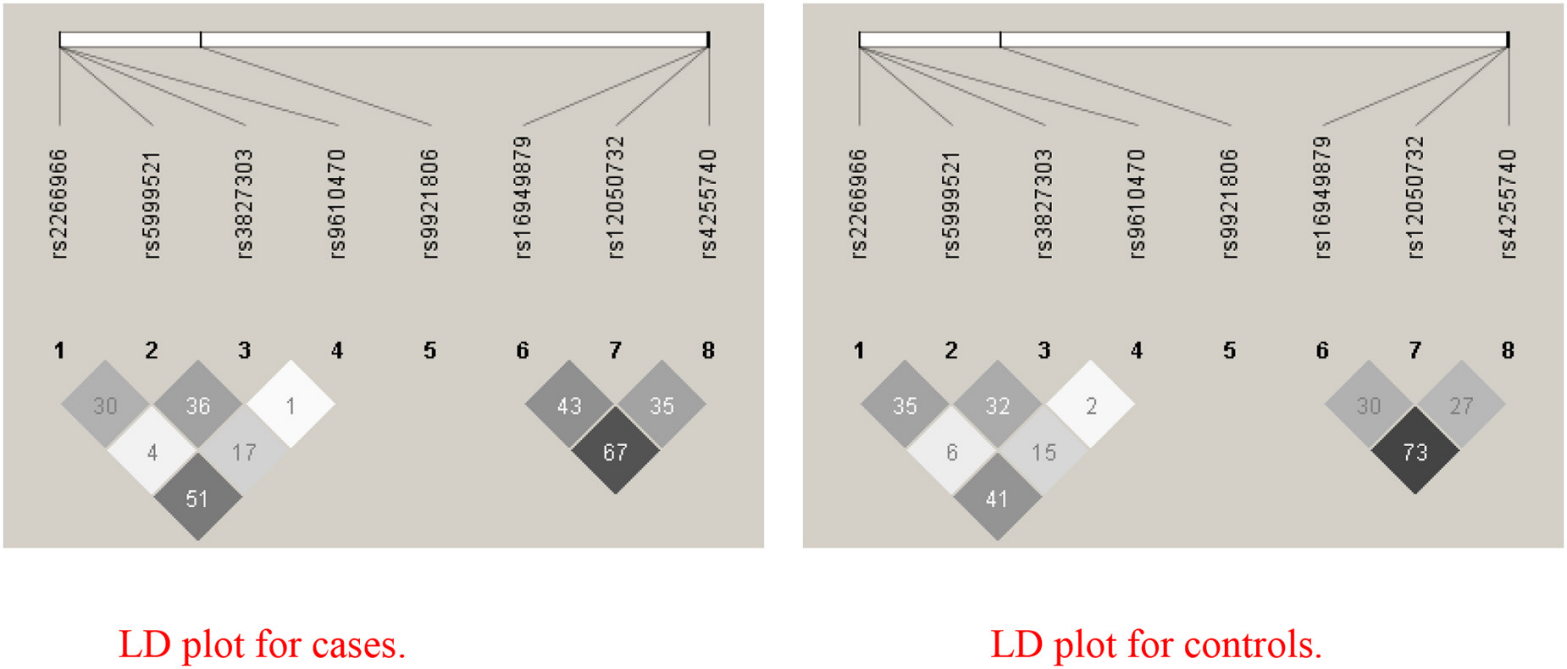
Linkage disequilibrium (LD) based haploview r^2^ plot

Table 2 shows the genotypes distributions of the 8 SNPs (rs12050732, rs16949879 and rs4255740 in MEK1; rs9921806 in ERK1; rs3827303, rs5999521, rs2266966 and rs9610470 in ERK2) and their association with PCOS. These 8 SNPs showed low pairwise LD each other in cases and controls, respectively (Figure 1). The *MEK1* rs12050732 SNP showed association with PCOS in the additive model for unadjusted data (OR=1.29 (95%CI: 1.06-1.58), *P* = 0.012). The *ERK2* rs5999521 SNP showed association with PCOS in the additive model for unadjusted data (OR=0.81 (95%CI: 0.67-0.99), *P*= 0.040). The ERK2 rs2266966 SNP showed association with PCOS in the additive model for unadjusted data (OR=0.66 (95%CI: 0.51-0.86), *P* = 0.002). In these all 3 polymorphisms, the data was insignificant when adjusted for age and BMI (*P*>0.05). The ERK2 rs9610470 SNP did marginal association with PCOS in recessive model (OR=0.16; 95%CI: 0.02-1.37; *P* = 0.096). The remaining 4 SNPs (rs16949879 and rs4255740 in MEK1; ERK1 rs9921806; rs3827303 in ERK2) showed no association with PCOS risk (Table 2).

**Table 2:** Association between MEK and ERK polymorphisms and PCOS risk

SNP	Genotype	Case	Control	OR (95%CI)	*P*	OR (95%CI)^a^	*P*^a^
*MEK1* rs12050732	AA	138	159	1.00	1.000	1.00	1.000
	CA	188	196	1.11(0.82-1.50)	0.518	1.06(0.73-1.56)	0.753
	CC	79	50	1.82(1.20-2.77)	**0.005^b^**	1.54(0.89-2.66)	0.124
	Dominant			1.25(0.94-1.67)	0.126	1.16(0.81-1.66)	0.431
	Recessive			1.72(1.17-2.53)	**0.006^b^**	1.49(0.90-2.46)	0.124
	Additive			1.29(1.06-1.58)	**0.012^b^**	1.19(0.92-1.55)	0.179

*MEK1* rs16949879	AA	140	142	1.00	1.000	1.00	1.000
	GA	173	173	1.01(0.74-1.39)	0.930	0.99(0.66-1.48)	0.963
	GG	75	68	1.12(0.75-1.67)	0.585	1.31(0.79-2.19)	0.293
	Dominant			1.04(0.78-1.40)	0.775	1.08(0.74-1.56)	0.700
	Recessive			1.11(0.77-1.60)	0.574	1.32(0.84-2.09)	0.233
	Additive			1.05(0.86-1.28)	0.621	1.12(0.87-1.44)	0.366

*MEK1* rs4255740	CC	176	174	1.00	1.000	1.00	1.000
	TC	189	188	0.99(0.74-1.33)	0.967	1.01(0.70-1.47)	0.953
	TT	39	43	0.90(0.55-1.45)	0.657	1.18(0.65-2.15)	0.585
	Dominant Dominant			0.98(0.74-1.29)	0.863	1.04(0.73-1.48)	0.824
	Recessive			0.90(0.57-1.42)	0.650	1.18(0.67-2.07)	0.578
	Additive			0.96(0.78-1.19)	0.733	1.06(0.81-1.39)	0.668

*ERK1* rs9921806	CC	261	282	1.00	1.000	1.00	1.000
	TC	128	109	1.27(0.93-1.72)	0.127	1.26(0.85-1.86)	0.255
	TT	13	14	1.00(0.46-2.17)	0.993	2.37(0.84-6.67)	0.103
	Dominant			1.24(0.92-1.66)	0.155	1.34(0.91-1.95)	0.134
	Recessive			0.93(0.43-2.01)	0.860	2.22(0.79-6.21)	0.129
	Additive			1.16(0.90-1.50)	0.244	1.35(0.97-1.88)	0.074

*ERK2* rs3827303	GG	258	265	1.00	1.000	1.00	1.000
	CG	132	121	1.12(0.83-1.51)	0.458	1.07(0.73-1.58)	0.737
	CC	15	18	0.86(0.42-1.74)	0.666	1.03(0.43-2.44)	0.949
	Dominant			1.09(0.81-1.45)	0.574	1.06(0.73-1.54)	0.745
	Recessive			0.82(0.41-1.66)	0.589	1.01(0.43-2.37)	0.986
	Additive			1.04(0.81-1.32)	0.775	1.05(0.77-1.42)	0.782

*ERK2* rs5999521	AA	162	139	1.00	1.000	1.00	1.000
	GA	183	185	0.85(0.63-1.15)	0.292	1.10(0.74-1.63)	0.638
	GG	60	79	0.65(0.43-0.98)	**0.038^b^**	0.72(0.43-1.20)	0.203
	Dominant			0.79(0.59-1.05)	0.106	0.97(0.67-1.41)	0.886
	Recessive			0.71(0.49-1.03)	0.072	0.68(0.43-1.08)	0.103
	Additive			0.81(0.67-0.99)	**0.040^b^**	0.88(0.69-1.13)	0.327

*ERK2* rs2266966	TT	279	241	1.00	1.000	1.00	1.000
	CT	121	150	0.70(0.52-0.94)	**0.016^b^**	0.92(0.63-1.33)	0.643
	CC	5	14	0.31(0.11-0.87)	**0.026^b^**	0.33(0.09-1.14)	0.079
	Dominant			0.66(0.50-0.89)	**0.005^b^**	0.86(0.60-1.23)	0.408
	Recessive			0.35(0.12-0.98)	**0.045^b^**	0.34(0.10-1.17)	0.086
	Additive			0.66(0.51-0.86)	**0.002 ^b^**	0.81(0.59-1.12)	0.208

*ERK2* rs9610470	TT	330	323	1.00	1.000	1.00	1.000
	CT	74	76	0.95(0.67-1.36)	0.791	1.22(0.79-1.9)	0.372
	CC	1	6	0.16(0.02-1.36)	0.094	0.21(0.02-2.00)	0.176
	Dominant			0.90(0.63-1.27)	0.534	1.13(0.73-1.74)	0.578
	Recessive			0.16(0.02-1.37)	0.096	0.21(0.02-1.91)	0.165
	Additive			0.85(0.61-1.17)	0.319	1.03(0.69-1.53)	0.886

^a^ Adjusted for age and BMI. ^b^ Significant parameters with *P* < 0.05 are represented in bold.

We also assessed the cumulative effects of three SNPs (MEK1 rs12050732, ERK2 rs5999521 and ERK2 rs2266966) on PCOS risk (Table 3). We observed allele-dosage association between the number of allele variants and PCOS risk in the additive model for unadjusted data (*P*_trend_ = 0.001) and marginal association when data was adjusted for age and BMI (*P*-value = 0.09). Individuals with 5-6 risk alleles showed higher risk for PCOS than those with 0–2 risk alleles for unadjusted data (OR = 1.23 (95% CI: 1.08-1.40), *P* = 0.001). But, the correlation was not significant when the data was adjusted for age and BMI (*P* = 0.88). Furthermore, we observed no significant association between the three SNPs and PCOS risk in subgroups stratified by age (≤ 30 y and > 30 y) and BMI (≤ 25 kg/m^2^ and > 25 kg/m^2^; data not shown).

**Table 3:** Association between combination of MEK1 rs12050732, ERK2 rs5999521 and ERK2 rs2266966 polymorphisms and PCOS risk

No. of risk alleles	case	control	OR (95% CI)	*P*	OR (95%CI)^a^	*P*^a^
0-2	82	110	1	1	1	1
3	73	91	1.08(0.71-1.64)	0.73	1.38(0.83-2.30)	0.22
4	113	107	1.19(0.98-1.45)	0.08	0.92(0.69-1.24)	0.59
5-6	137	98	1.23(1.08-1.40)	**0.001^b^**	1.02(0.83-1.24)	0.88
Trend			1.24(1.10-1.41)	**0.001^b^**	1.14(0.98-1.33)	0.09

^a^ Adjusted for age and BMI. ^b^ Significant parameters with *P* < 0.05 are represented in bold.

## DISCUSSION

In this case-control study of Chinese individuals, we evaluated the association of genetic variants identified by RegulomeDB in MAPK/ERK genes with PCOS susceptibility. We found that MEK1 rs12050732, ERK2 rs2266966 and ERK2 rs5999521 were associated with PCOS risk.

The MAPK/ERK pathway plays an important role in the pathogenesis of PCOS. The phospho-ERK1/2 levels are reduced in PCOS granulosa cells [22], whereas the MAPK/ERK signaling pathway is hyperactive in the PCOS endometrium [23]. PCOS patients showed elevated serum T and LH levels [3]. In PCOS patients, MAPK signaling was activated by the LH receptor [24, 25]. Moreover, T levels were elevated when patients were treated with the MAPK kinase inhibitor PD98059 [26, 27]. These data suggest that MAPK/ERK signaling regulates T and LH levels in PCOS patients [24-27].

MEK1 is located on human chromosome 15q22.31 and is a key gene in MAPK pathway [10]. LH consists of α and β subunits, of which the β-subunit synthesis is the rate-limiting step in LH synthesis [28]. Overexpression of constitutively active MEK1 activates the LH β-subunit promoter [24]. MEK1 inhibitor U0126 blocks androgen-induced DNA synthesis, thereby demonstrating the role of MEK1 in androgen metabolism [29]. MEK1 rs12050732 is found in patients with esophageal carcinoma and gastric cancer [30]. MEK1 rs12050732 is a cis-eQTL for DIS3L, which has a RNA exosome subunit with endonucleolytic and 3’-5’exonucleolytic activity that degrades RNA [31].

ERK2 is located on chromosome 22q11.22 and is involved in many critical signal transduction pathways including LH signaling during ovulation [32, 33]. ERK2 deletion in mouse granulosa cells disrupts LH-induced meiotic progression in oocytes, ovulation, and luteinization [34]. ERK2 activation is critical for the normal feedback mechanism of insulin signaling and its aberrant activation contributes to insulin resistance in PCOS [14, 35]. The RegulomeDB score of both the ERK2 SNPs, rs2266966 and rs5999521 was 2b, which indicated binding to many proteins including transcription factors such as CTCF, IRF4, MYC, STAT2, and SP1 [36].

MEK2 is located on chromosome 19p13.3 and phosphorylates MAPK1/ERK2 and MAPK2/ERK3, thereby activating them [37]. RegulomeDB showed only one potentially functional variant of MEK2, which could not be genotyped. ERK1 is located on chromosome 16p11.2 and translocates to the nucleus when activated by upstream kinases [37].

In summary, our study shows that multiple polymorphisms in *MAPK/ERK* genes are associated with PCOS risk in Chinese women. Further independent studies with large sample sizes are necessary to unravel the molecular mechanisms underlying this association and to further confirm our findings.

## MATERIALS AND METHODS

### Study population

This study was approved by the Human Research Ethics Committee of Changzhou Maternity and Child Health Care Hospital Affiliated to Nanjing Medical University. We recruited 405 PCOS and control subjects each for this study as described previously [38]. All the study subjects were Chinese women of Han ethnicity. They were recruited from the Department of Obstetrics and Gynecology of the Affiliated Hospital of Medical School of Nanjing University, the Centre of Reproduction Memorial Hospital of Sun Yat-Sen University, and the Department of Obstetrics and Gynecology of Anhui Medical University.

### PCOS diagnosis

PCOS was diagnosed according to the revised 2003 consensus on diagnostic criteria and long-term health risks related to PCOS [39]. The criteria for PCOS diagnosis were as follows: (1) oligo ovulation and /or anovulation; (2) clinical and /or biochemical characterization of hyperandrogenism and (3) polycystic ovarian morphology based on ultrasound. At least 2 of the 3 criteria needed to be positive for the case to be classified as PCOS. Moreover, patients with diseases that cause hyperandrogenism such as congenital adrenal hyperplasia, hypothyroidism, Cushing’s syndrome and androgen-secreting tumors were excluded. The study subjects had not received hormone drugs or oral contraceptives three months prior to analysis. All subjects were interviewed face-to-face to collect demographic data and exposure information by trained interviewers. After signing informed consent, peripheral blood was collected during the 3^rd^, 4^th^ and 5^th^ day of the menstrual cycle and at any time for those who had amenorrhea. Serum levels of FSH, LH, T and estradiol (E2) were measured by RIA assay (Beijing North Institute of Biological Technology of China and the CIS Company of France).

### Polymorphism genotyping

We identified all polymorphisms in the *MEK1*, *MEK2*, *ERK1* and *ERK2* genes by Genome Reference Consortium Human Build 37 patch release 13 (GRCh37/hg19) assembly. A lower RegulomeDB score indicated strong probability for a variant to be located in the functional region of a gene. Therefore, a polymorphism with a score of 1 probably affected transcription and expression of a target gene. We selected SNPs that were classified in the 1-2b category (http://regulome.stanford.edu). The selection criteria included minor allele frequency (MAF) ≥ 0.05 and Hardy–Weinberg equilibrium (HWE) ≥ 0.05. Finally, SNPs with R^2^ values of pairwise linkage disequilibrium (LD) < 0.8 were selected. The genomic DNA isolation method was as described previously [38]. Briefly, genomic DNA was isolated by proteinase K digestion and phenol chloroform extraction from leukocytes isolated from venous blood. Genotyping was performed by Sequenom’s MassARRAY® iPLEX assay according to manufacturer’s instructions without knowing the status of case and control. We included duplicate samples (5%) for internal consistency and three water blanks as negative control in each reaction plate. Eight SNPs were successfully genotyped with call rate > 95% (Table 2).

### Statistical analysis

We used χ^2^ test for categorical variables and Student's t-test for continuous variables to evaluate the differences between the cases and controls. HWE was evaluated using the goodness-of-fit χ^2^ test among controls. The association between genotypes and PCOS risk with or without adjustments for age and BMI was performed by calculating odds ratios (ORs) and their 95% confidence intervals (CIs) from logistic regression analyses. The statistical analysis was conducted using SPSS Statistics Version 17.0 or PLINK (http://www.cog-genomics.org/plink2/) software.

## Abbreviations

MAP: mitogen-activated protein
PCOS: polycystic ovarian syndrome
SNPs: single nucleotide polymorphisms
BMI: body mass index
FSH: follicle-stimulating hormone
LH: luteinizing hormone
E2: estradiol
T: total testosterone
GWAS: genome-wide association studies
MAPKs: MAP kinases
ERKs: extracellular signal-regulated kinases
